# Effectiveness of compression stockings to prevent the post-thrombotic syndrome (The SOX Trial and Bio-SOX biomarker substudy): a randomized controlled trial

**DOI:** 10.1186/1471-2261-7-21

**Published:** 2007-07-24

**Authors:** Susan R Kahn, Hadia Shbaklo, Stan Shapiro, Philip S Wells, Michael J Kovacs, Marc A Rodger, David R Anderson, Jeffrey S Ginsberg, Mira Johri, Vicky Tagalakis, the SOX Trial Investigators

**Affiliations:** 1 Centre for Clinical Epidemiology and Community Studies, Jewish General Hospital, Montreal, Quebec, Canada; 2 Department of Epidemiology and Biostatistics, McGill University, Montreal, Quebec, Canada; 3 Division of Hematology, The Ottawa Hospital, Ottawa, Ontario, Canada; 4 Thrombosis Unit, London Health Sciences Centre, London, Ontario, Canada; 5 Thrombosis Program, Division of Hematology, University of Ottawa, Ottawa, Ontario, Canada; 6 Clinical Epidemiology Program, The Ottawa Hospital-General Campus, Ottawa, Ontario, Canada; 7 Thrombosis Unit, Queen Elizabeth II Health Sciences Centre, Halifax, Nova Scotia, Canada; 8 Department of Medicine, McMaster University Medical Center, Hamilton, Ontario, Canada; 9 Health Administration Department, Université de Montréal, Montreal, Quebec, Canada

## Abstract

**Background:**

Post thrombotic syndrome (PTS) is a burdensome and costly complication of deep venous thrombosis (DVT) that develops in 20–40% of patients within 1–2 years after symptomatic DVT. Affected patients have chronic leg pain and swelling and may develop ulcers. Venous valve disruption from the thrombus itself or thrombus-associated mediators of inflammation is considered to be a key initiating event for the development of venous hypertension that often underlies PTS. As existing treatments for PTS are extremely limited, strategies that focus on preventing the development of PTS in patients with DVT are more likely to be effective and cost-effective in reducing its burden. Elastic compression stockings (ECS) could be helpful in preventing PTS; however, data on their effectiveness are scarce and conflicting.

**Methods/Design:**

The SOX Trial is a randomized, allocation concealed, double-blind multicenter clinical trial. The objective of the study is to evaluate ECS to prevent PTS. A total of 800 patients with proximal DVT will be randomized to one of 2 treatment groups: ECS or placebo (inactive) stockings worn on the DVT-affected leg daily for 2 years. The primary outcome is the incidence of PTS during follow-up. Secondary outcomes are severity of PTS, venous thromboembolism (VTE) recurrence, death from VTE, quality of life and cost-effectiveness. Outcomes will be evaluated during 6 clinic visits and 2 telephone follow ups. At baseline, 1 and 6 months, blood samples will be obtained to evaluate the role of inflammatory mediators and genetic markers of thrombophilia in the development of PTS (Bio-SOX substudy).

**Discussion:**

The SOX Trial will be the largest study and the first with a placebo control to evaluate the effectiveness of ECS to prevent PTS. It is designed to provide definitive data on the effects of ECS on the occurrence and severity of PTS, as well as DVT recurrence, cost-effectiveness and quality of life. This study will also prospectively evaluate the predictive role of biomarkers that are reflective of putative underlying pathophysiological mechanisms in the development of clinical PTS. As such, our results will impact directly on the care of patients with DVT.

**Trial Registration:**

NCT00143598 and ISRCTN71334751

## Background

### The post thrombotic syndrome

The post thrombotic syndrome (PTS) (sometimes called 'post-phlebitic syndrome') is a chronic condition that develops in 20–40% of patients within 1–2 years after symptomatic deep venous thrombosis (DVT). A severe form, which can include venous ulcers, affects 1/4 to 1/3 of patients with PTS [1]. In contrast to research gains in the areas of diagnosis, prevention and treatment of acute venous thromboembolism (VTE), PTS has been understudied and as a result, treatment options are limited.

Patients with PTS experience pain, heaviness, swelling, or other symptoms in the affected limb, which are typically aggravated by standing or walking and improve with rest and recumbency. Edema, venous ectasia, hyperpigmentation, eczema, and varicose collateral veins may be apparent. In severe cases, ulceration can occur [2].

Since PTS is a direct consequence of DVT, its prevalence is influenced by the incidence of DVT. Despite advances in VTE prevention and treatment, the annual incidence of VTE has not decreased over time, and remains at 1.0–1.6 per 1000 persons per year, with a per-person lifetime incidence of 2–5% [3,4]. Because of its prevalence and chronicity, PTS is expensive, both in terms of direct medical costs and indirect costs such as loss of productivity and thus it is burdensome to patients and society [5].

### Risk factors for the development of PTS

While hereditary and acquired risk factors that predispose to the development of VTE are widely known [6], factors that influence the development of PTS after DVT have not been well elucidated. The only clearly identified clinical risk factor for PTS is recurrent, ipsilateral DVT [7,8]. The site (proximal vs. distal), size and occlusiveness of the initial DVT and the intensity (i.e. target INR) of long term anticoagulation for DVT do not appear to reliably predict PTS [1,7-10]. It is not clear why some patients develop PTS while others recover from their DVT. As a result, physicians are unable to provide their DVT patients with reliable, individualized prognostic information.

### Potential role of inflammation, d-Dimer and thrombophilia in the post-thrombotic syndrome

While the pathophysiology of PTS is incompletely understood, it is likely that the acute thrombus itself, associated mediators of inflammation, and the process of vein recanalization in the weeks following DVT induce damage to venous valves, leading to valvular incompetence (reflux). Valvular incompetence and/or persistent venous obstruction by thrombus cause venous hypertension, which promotes capillary leakage of plasma proteins, erythrocytes and leukocytes and the development of venous ectasia and varicosities. The result is edema, tissue hypoxia, and ultimately, in some cases, skin ulceration [11-14].

Inflammation and thrombosis are closely interrelated [15-17]. It has been appreciated over the last few years that arterial atherothrombosis is, at least in part, an inflammatory disease, and that elevations in markers of inflammation, such as C-reactive protein (CRP), increase the risk of future clinical cardiovascular events [18]. Clinically, patients with DVT exhibit cardinal signs of inflammation such as redness, warmth, swelling, pain and fever. Several clinical studies have examined the association between levels of inflammatory markers and venous thrombosis, and found that a two- to six-fold increase in the risk of DVT associated with elevations in plasma levels of CRP, interleukin (IL)-6, IL-8, monocyte chemotactic protein (MCP)-1 or tumor necrosis factor (TNF)-alpha [19]. A recent paper showed that CRP is elevated in patients with acute DVT (median 37.5 mg/L) compared with controls (5.0 pg/L), and that levels decline during the first 5 days of DVT treatment. Similar trends were noted for IL-6 [20], leading the authors to conclude that the thrombotic process produces a systemic inflammatory response and to speculate whether the observed decrease in levels was partly caused by treatment with heparin, which is known to have anti-inflammatory properties distinct from its anticoagulant properties [21]. Whether levels of markers of inflammation are predictive of PTS has not previously been examined.

D-dimer is a degradation product of cross-linked fibrin that reflects fibrinolysis and is an indirect marker of coagulation activation. Recent work performed by our group and by others suggests that D-dimer levels appear to be a significant predictor of first VTE [22] and of recurrent VTE [23-28], whether measured during or after anticoagulation. Independent of VTE recurrence, persistent coagulation activation, as reflected by elevated D-dimer levels, may also predict a higher risk of developing PTS after DVT [29].

Thrombophilia refers to an inherited or acquired predisposition to VTE. Over 10% of the general population is affected by one or more identifiable inherited thrombophilias which have been shown to underlie at least 1/3 of cases of VTE [30]. The most common inherited thrombophilias are the single nucleotide polymorphisms Factor V Leiden, which renders coagulation factor V resistant to the anticoagulant effects of activated protein C [31], and Prothrombin G20210A, which leads to elevated plasma prothrombin levels [32,33]. Recent studies have also established that elevation of Factor VIII level is an inherited risk factor both for first and for recurrent VTE [34-36], independent of age, sex, Factor V Leiden, Prothrombin G20210A or markers of acute phase activation such as CRP [35,37,38].

While results of a few studies to date suggest that thrombophilia does not increase the risk of developing PTS [7,8,10], not all thrombophilias have been examined and interactions among disorders have not been studied. Further investigation of the potential link between thrombophilia and PTS is warranted.

### Management of PTS

PTS could be averted by primary prevention of the initial DVT with the judicious use of thromboprophylaxis [39], and by preventing recurrent ipsilateral DVT by prescribing adequate anticoagulation for the initial DVT [40]. However, at least 1/2 of all cases of DVT occur unpredictably, hence are not preventable [41,42]. There is no definitive evidence that using thrombolysis to treat DVT reduces the incidence of PTS [43]. The treatment of established PTS is limited and frustrating for patients. Severe PTS can be managed with long-term use of an intermittent compression extremity pump [44,45]. Management of venous ulcers is labor intensive and protracted, and involves compression therapy, leg elevation, topical dressings, and sometimes surgery [2,46]. Ulcers are often recalcitrant to treatment, and tend to recur [47].

A significant reduction in the overall burden of PTS is unlikely to be achieved by attempts to prevent the initial DVT or by treatment of established PTS. Rather, strategies that focus on preventing the development of PTS in patients with DVT are more likely to be effective and cost-effective in reducing the patient and societal impact of PTS.

### Elastic compression stockings for the prevention of PTS

Graduated elastic compression stockings (ECS) work by providing graded compression to the leg that is highest at the ankle, which assists the calf muscle pump, reduces venous hypertension and valvular reflux, and consequently reduces edema, improves tissue microcirculation, and prevents skin breakdown [48,49]. Both knee-length and thigh-length stockings appear to have equal physiological effects, but knee-length ECS are easier to apply and are more comfortable [50]. The effectiveness of daily use of ECS to prevent PTS is supported by two studies [8,51] but challenged by another [52]. All three studies have limitations that could affect their validity and generalizability. The first trial, Brandjes' study of 194 patients with symptomatic proximal DVT, provides evidence supporting the effectiveness of ECS. Patients were randomized to daily use of custom-made, knee-length ECS applied within 2–3 weeks of diagnosis for at least 2 years (class II compression, i.e. 30–40 mm Hg pressure at the ankle), or no stocking. Use of ECS resulted in a 50% reduction in the incidence of PTS, diagnosed using a modification of Villalta's clinical PTS scale [53], or an absolute decrease from 47% to 20% of mild/moderate PTS and from 23% to 11% of severe PTS [51]. In contrast, a randomized trial conducted by Ginsberg suggested that ECS were not of benefit in preventing PTS [52]. A strength of Ginsberg's study was the use of a control comparison group that wore sham stockings (i.e. stockings that were 1–2 sizes too big to be effective). However, because of the small number of patients with PTS, benefit or harm of up to 30% could not be definitively excluded. Recently, Prandoni published the results of a trial performed at a single center in Italy to evaluate the effectiveness of ECS to prevent PTS [8]. Among 180 patients with proximal DVT, those randomized to wear daily ECS had a 50% reduction in the rate of PTS after a 2 year period, compared with controls. This study, like Brandjes', lacked a placebo control, an important limitation due to the subjective nature of many of the components of the standardized scale that was used to diagnose PTS [54]. As the Prandoni study was the first, single positive study of "off-the-rack" elastic compression stockings, it requires replication. Also, as the study was conducted entirely at a single center in Italy, further evaluation of the generalizability of this data to North American clinical practice in a multi-center study is needed.

In light of the above, we believe that a large scale, randomized placebo-controlled trial of ECS to prevent PTS is needed to provide definitive evidence of effectiveness, or lack of effectiveness, of ECS. This will allow physicians to make informed, evidence-based decisions regarding their use in DVT patients. Furthermore, such a trial will permit prospective evaluation of the predictive role of markers of inflammation, d-Dimer and thrombophilia in PTS.

## Methods/Design

The SOX Trial is a Canadian, multicenter, randomized double-blind controlled trial in patients with a first episode of proximal DVT.

### Aims of the study

The primary aim of the study is to evaluate whether graduated elastic compression stockings compared to inactive (placebo) stockings, worn daily for 2 years after DVT is diagnosed, decrease the incidence of PTS.

Secondary aims are 1) to evaluate whether active compared with inactive stockings reduce the severity of PTS, 2) to compare mean quality of life (QoL) scores during follow-up in the active intervention vs. control groups, 3) to describe the rates of recurrent DVT, death from VTE, venous ulcers, and major bleeding during the 2-year follow-up in the active intervention vs. control groups, 4) to evaluate the cost-effectiveness of ECS for the prevention of PTS, and 5) to determine whether markers of inflammation, D-dimer and thrombophilia influence the development and severity of PTS (Bio-SOX biomarker substudy).

### Trial design and description of the intervention

#### Original trial design

The study was initially designed as a factorial design randomized clinical trial whose primary aims were to determine whether (1) elastic compression stockings used for 2 years compared to inactive (placebo) stockings, and (2) celecoxib, a COX-II inhibitor, used for 30 days compared to placebo, were effective in preventing PTS in patients with symptomatic proximal DVT. The rationale for the use of a COX-II inhibitor was to assess whether an anti-inflammatory drug used acutely could limit the extent of venous valvular damage and reduce the frequency of subsequent PTS. However, in December 2004, The National Institutes of Health announced suspension of the use of celecoxib for all participants in a large colorectal cancer prevention clinical trial conducted by the National Cancer Institute, because analysis by an independent Data Safety and Monitoring Board (DSMB) showed a 2.5-fold increased risk of major fatal and non-fatal cardiovascular events for participants taking the drug compared to those on a placebo. As a result of this announcement, the SOX Trial Steering Committee decided on December 21, 2004 to discontinue the Celecoxib intervention of the SOX Trial [55]. This decision was made after independent meetings of the Steering Committee and the SOX Trial DSMB and was based on patient safety and also on study feasibility. The SOX Trial thus resumed in February 2005 as a parallel group randomized trial, i.e. without the celecoxib/placebo intervention.

#### Current trial design

**Figure 1:**
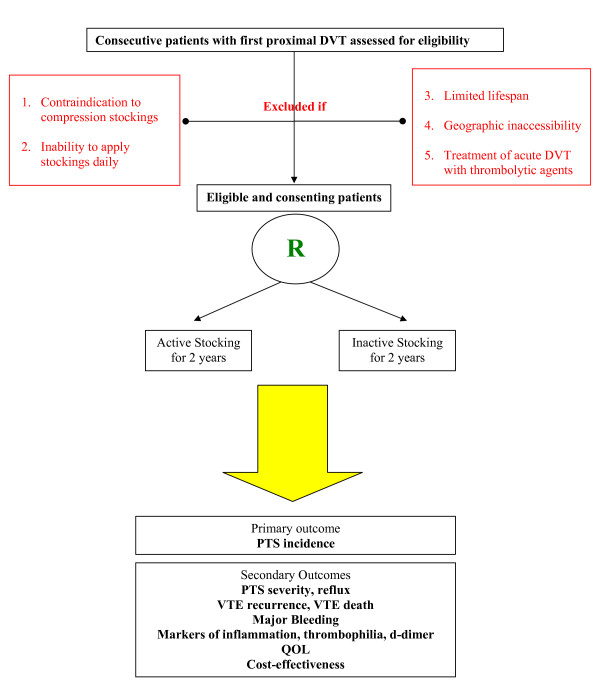
Study architecture-Flow diagram: The SOX Trial.

The study is a randomized, allocation concealed, double-blind multicenter clinical trial with an intervention allocation ratio of 1:1 (figure 1 summarizes the study design). Once a patient is deemed eligible and consents to the study, the research nurse at each site logs onto a web-based interface which allocates the patient to one of 2 treatment groups: active stockings or inactive (placebo) stockings. Treatment group is assigned randomly using permuted blocks of randomly varying sizes at each study site to maintain close balance of the numbers in each treatment group, at any time during the trial, and to ensure allocation concealment. The active stockings intervention consists of knee-length, 30–40 mm Hg (Class II), graduated ECS worn on the DVT-affected leg daily, applied upon waking and removed upon retiring, beginning as early as possible within 14 days after DVT diagnosis and continued for 2 years. The inactive stockings intervention (the control intervention) consists of knee-length, inactive stocking (5 mm Hg compression at ankle; similar to store bought trouser sock), identical in appearance to the active stockings, worn on the DVT-affected leg daily, applied upon waking and removed upon retiring, beginning as early as possible within 14 days weeks after DVT diagnosis, and continued for 2 years. Active and inactive stockings are manufactured by SIGVARIS Corp.

#### Patient eligibility criteria

Patients presenting with a first, symptomatic, objectively confirmed proximal DVT diagnosed within the last 10 days (with or without concurrent distal DVT or pulmonary embolism), who have no contraindications to standard anticoagulant therapy and who provide informed consent are eligible for the study.

Patients are excluded if they 1) have a contraindication to compression stockings e.g. previously documented moderate to severe peripheral arterial disease of the lower extremities, absence of palpable pedal pulses or arterial compromise due to massive venous obstruction; 2) have a limited lifespan (estimated < 6 months); 3) have geographic inaccessibility preventing their return for follow-up visits; 4) demonstrate inability to apply stockings daily (e.g. severe arthritis, arm paralysis, and unavailability of a caregiver to apply stockings daily); or 5) were prescribed lytic therapy to treat their acute DVT.

#### Study procedures

Once the patient is recruited and randomized via the SOX Trial's web-based interface, the computer generates a unique treatment code for each patient, according to treatment group and center. The study nurse faxes the patient's leg measurements to the stocking supplier, who then ships the assigned pair of ECS to the patient (i.e. correct size, according to patient's measurements; active or inactive stocking, according to patient's treatment code). ECS are pre-packaged by the manufacturer in a plain box, labeled with the unique stocking code (i.e. patient and health personnel blinded). The same procedure is used to replace the patient's ECS every 6 months during the trial.

Several strategies are used to protect against bias, including: randomization with allocation concealment, enrolling consecutive patients, blinding subjects to treatment assignment, blinding investigators, study nurses and outcome assessors (e.g. vascular technicians, radiologists) to treatment assignment, strict inclusion and exclusion criteria, and use of validated measures to diagnose PTS, recurrent VTE and to measure QoL. To prevent unblinding of study nurses, patients are instructed not to wear ECS on the day of their study visits. While it is possible that patients may be able to distinguish active from inactive ECS, this is minimized by (1) using inactive ECS, developed by the manufacturer, that appear identical to active ECS, and (2) recruiting patients with a first DVT, i.e. likely to be "ECS-naïve". In addition to such pre-trial design features, we will assess the blinding procedures by asking patients, investigators and research nurses at the end of the trial (i.e. at 2 years or at time of study termination) if they were aware of the treatment assignment.

Study patients are followed for 2 years after enrollment. The procedures at each visit are shown in Table 1. At any time during the study, if a patient has symptoms or signs suggestive of recurrent VTE, he contacts the study team for medical assessment, at which time recurrent VTE will be ruled in or ruled out. Similarly, if an adverse event occurs, the patient contacts the study team for evaluation. If a patient withdraws from the study, a Study Termination form is completed to account for withdrawal, which will document the reason(s) for withdrawal. If a patient dies, a Death Form is completed which documents the cause(s) of death, further classified as VTE likely or unlikely. Throughout study follow-up, co-interventions are recorded (e.g. diuretics, analgesics, exercise). While not encouraged, any temporary use of elastic bandages/wraps or active stockings during the acute phase of DVT is systematically tracked.

**Table 1:** Details of study visits and telephone calls

***Enrollment visit***
• The nurse completes the baseline form (inclusion/exclusion criteria, demographic and clinical variables, results of objective tests for DVT), PTS scale and pain scale
• The patient self-completes the QOL form
• The nurse performs the leg measurements to size the stockings
• The nurse teaches patients how to apply (using a plastic leg model) and care for stockings (handling, washing), and reinforces the importance of daily use
• Study stockings are supplied to the patient (within a few days after enrollment)
• A venous blood sample is taken for markers of inflammation
**2-week telephone call**
• The nurse reinforces the importance of wearing stockings daily
• Bleeding/Other Adverse Event form and pain scale are completed
For the following visits, the patient does not wear study stocking to prevent unblinding of study nurse:
**1-month visit**
• The nurse completes the follow-up form (includes data on symptoms and signs of PTS, suspected recurrent VTE, compliance with stockings, pain scale)
• The patient self-completes the QOL form
• Bleeding/Other Serious Adverse Event form is completed
• A venous blood sample is taken for markers of inflammation and genetic thrombophilia
• The nurse reinforces the importance of wearing stockings daily
**2-month telephone call**
• Same as the 2-week telephone call
**6-month visit**
• The nurse completes the follow-up form
• The patient self-completes the QOL form
• Bleeding/Other Serious Adverse Event form is completed
• Leg measurements are taken, and a new pair of stockings is supplied
• A venous blood sample is taken for markers of inflammation, d-dimer and coagulation-based assays
• The nurse reinforces the importance of wearing stockings daily
**1-year visit**
• Same as 6-month visit except no blood sampling
• A venous ultrasound is performed to assess reflux
**18-month visit**
• Same as 6-month visit except no blood sampling
**2-year visit**
• The nurse completes the follow-up form
• The patient self-completes the QOL form
• Bleeding/Other Serious Adverse Event form is completed
• The success of blinding (patient, nurse, and investigator) is assessed (or at Study Termination if earlier than 2 years).

### Primary and secondary outcome measures

The primary outcome measure is the incidence of PTS during 2 year follow up. PTS will be diagnosed at the 6 month visit or later if the patient reports pain and swelling of ≥ 1 month's duration that is typical in character (worse at the end of the day or with prolonged sitting or standing, and better after a night's rest and leg elevation), and that is present ≥ 6 months after the acute DVT [52]. Patients diagnosed with PTS will be considered to have reached the study endpoint. Severity of PTS will be graded according to the Villalta scale [53], a clinical post-thrombotic syndrome measure that rates the severity of five patient-rated symptoms and six clinician-rated clinical signs [53]. A Villalta score of 5–9 represents mild post-thrombotic syndrome; 10–14, moderate post-thrombotic syndrome; and > 15 or presence of a venous ulcer, severe post-thrombotic syndrome [53].

Secondary outcomes measures include:

• Reflux: Reflux will be assessed and quantified in a standardized fashion by venous ultrasound [52,56].

• Recurrent VTE: Objective tests and validated algorithms will be used to diagnose recurrent DVT and PE, as described previously [57-59], and all events and deaths will be independently and blindly adjudicated. Death from VTE will be assessed as 'likely' or 'unlikely' after review of the death certificate, patient chart, autopsy report (where available) and by contacting the treating physician.

• Bleeding: Clinically suspected bleeding events will be documented by recording the clinical event and results of any objective diagnostic testing performed. Bleeding will be defined as major if it is clinically overt and associated with a fall in hemoglobin of 20 g/L or a need for transfusion of ≥ 2 units of red blood cells; if it was intracranial or retroperitoneal; or if it warranted the permanent discontinuation of anticoagulation. Less severe clinically overt bleeding which is considered abnormal will be classified as minor.

• Markers of inflammation: C-reactive protein will be assayed using a nephelometric assay. IL-6, IL-8 and MCP-1 will be measured using commercial ELISA kits which include plasma controls (Biosource Europe SA, Nivelles, Belgium). At the time of blood draw for markers of inflammation, data will be recorded on time of day, type and duration of heparin use, concomitant medications and medical conditions.

• DNA polymorphisms associated with thrombophilia: QIAGEN kits and standard protocols will be used to extract DNA from peripheral blood leukocytes. Samples will be run on the ABI Prism 3100 Genetic Analyzer to analyze Factor V Leiden, Prothrombin G20210A, PAI-1 4G/4G, TAFI T1053C, TFPI C536T, TFPI T33C and FXIII Val34Leu.

• Coagulation and ELISA-based assays: Factor VIII assays will be performed using the PTT based Diagnostica Stago (Abbott Diagnostics Canada, Mississauga). D-dimer testing will be performed with the IL-Test Latex Agglutination method (Instrumentation Laboratory, Lexington, USA). Lupus anticoagulant testing will be performed on an ACL9000 (Instrumentation Laboratory, Lexington, USA). Standard aPTTs are performed using Alexin HS (Trinity Biotech USA, St. Louis, USA) and HemosIL APTT-SP (Instrumentation Laboratory, Lexington, USA) as a screening test. Abnormal results are followed by a 50:50 mix using Cryocheck Normal (Precision Biologic, Dartmouth, NS), followed by Platelet Neutralization Procedure. Additionally, a Dilute Russell Viper Venom test is performed using IL test LAC Screen followed by Confirm (Instrumentation Laboratory, Lexington, MA, USA). IgG and IgM antiphospholipid antibodies are tested usingapHL-HRP ELISA Kit (Louisville APL Diagnostics, Doraville, USA).

• Quality of life measures: Quality of life will be measured using the SF-36 questionnaire for generic QOL [60,61]., and the VEINES-QOL/Sym questionnaire for venous disease-specific quality of life, which our group developed and validated [62,63]

• Cost Effectiveness measures: A decision tree-based cost effectiveness analysis will be used to assess the relative cost-effectiveness of the active stockings in reducing the incidence of PTS, as compared to a strategy of inactive stockings [64,65]. Tree structure will schematically reflect the clinical trajectory for each strategy [66]. Sensitivity analyses will be conducted to verify the robustness of the qualitative conclusions to variations in the data [67].

### Sample size and power calculations

We hypothesized that event rates (i.e. PTS at 2 years) will be 30% in the inactive stocking vs. 20% in the active stocking group, i.e. a risk reduction of 33%. Our baseline event rate and effect size for the stocking group were based on a systematic review of available published data [68]. The total sample size required to detect this difference in event rates with a two-tailed α of 0.05 and 80% power is 600 [69]. We increased our sample size to 800 to take into account a projected 25% loss-to-follow up by 2 years.

For the biomarker analyses, our sample size provides > 80% power to detect ORs of 0.5 or lower, or 2.0 or higher. Larger alternatives will be associated with increased power. With regard to inflammatory markers, the analysis will be largely exploratory, as this study will be among the first to provide prospective data on levels of markers of inflammation in patients with DVT and PTS. However, the study design provides power of at least 80% to detect differences on the order of 1/3 of a standard deviation or larger between initial values of inflammatory markers in the two allocation groups.

### Statistical analysis

An intention-to-treat analytical approach will be used for all outcomes. Descriptive statistics for baseline variables will be calculated to describe the baseline status of the treatment groups. For the primary outcome variable PTS, a logistic regression analysis initially adjusted only for center will be used to compare the incidence rates between the active vs. inactive stockings groups. In secondary analyses, other covariates that will be adjusted for are duration of anticoagulation with warfarin, recurrent ipsilateral DVT during follow-up, temporary use of active stockings, initial type and duration of heparin anticoagulation, co-morbid conditions that could lead to leg symptoms/signs and differences in pertinent baseline variables. The number needed to treat for benefit will be calculated. As an adjunct to the primary analysis, a secondary explanatory (per-protocol) analysis will be performed as well. Subgroup analyses by age, sex, level of compliance, duration of warfarin anticoagulation and recurrent VTE during study follow-up are also planned.

For the secondary outcomes valvular reflux, recurrent VTE, death from VTE, venous ulcer, major bleeding, and severity of PTS, rates in the two intervention groups will be described, and between-group differences explored in similar regression procedures. For differences in mean quality of life scores between intervention groups, analysis of variance incorporating treatment and center will be used. A difference of 4–5 points between groups is considered to be clinically meaningful. Analysis of covariance with adjustment for age, sex and comorbidity will also be used to compare quality of life between groups. For the biomarker variables, univariate analysis will be used to determine the strength of association between each biomarker variable and the occurrence of PTS. For genetic markers, heterozygotes and homozygotes will be analyzed together, given the rarity of homozygotes. IgG, IgM and lupus anticoagulant results will be dichotomized as normal or abnormal. Factor VIII, D-dimer and markers of inflammation will be analyzed both as continuous data and as proportion of values exceeding the upper 90^th ^percentile. For binary and nominal data, the appropriate chi-square tests will be used to examine the respective crude associations, and for continuous variables, the unpaired 2-tailed t-test. To examine adjusted associations, multiple logistic regression analyses will be performed in which the dependent variable is the presence of PTS. The independent variables to be tested are markers of thrombophilia, D-dimer, and levels of CRP, IL-6, IL-8 and MCP-1. Other variables of interest (for interaction and/or confounding) are age, sex, ethnicity, comorbid illness, allocation to active ECS, type and duration of heparin and warfarin use, type of DVT (unprovoked vs. secondary; anatomic extent of DVT), and recurrent DVT during follow-up. The analyses will aim to (1) identify the strength of the association between relevant biomarkers, other relevant explanatory variables and PTS; and (2) explore relevant interactions. Similarly, multiple linear regression will be performed in which the dependent variable is the severity of PTS, as measured by the Villalta scale.

An interim analysis for efficacy will be performed when half of the subjects have completed 2 years of follow-up. The analysis plans call for a Lan-DeMets alpha spending approach with an O'Brien Fleming boundary [70].

### Data management

All data are collected using standardized case report forms. Data is entered on-line at study sites using a customized web-based data entry tool, and data quality is maintained via use of validation checks at the time of data entry. Data will be reviewed and cleaned by the database coordinator on an on-going basis by initiating and following up on queries to the sites. Data management is being overseen by TrialStat^® ^(Ottawa, Canada). Analysis of the cleaned database will be carried out at the coordinating center under the supervision of study principal investigators.

### Trial committees

The Steering Committee is chaired by the study principal investigator and includes the trial coordinator and all SOX Trial grant co-applicants. The Steering Committee meets regularly and on an as-needed basis to monitor trial progress, assess the need for changes in procedures, and address issues that could affect the integrity or projected timeframe of the trial. The Expert Adjudication Committee for all outcome events is comprised of two thrombosis experts who are not co-investigators or collaborators. Clinical details and diagnostic studies relating to suspected outcome events and deaths are reviewed by the committee, who are blinded to treatment assignment. The Independent Data Safety and Monitoring Committee consists of an experienced thrombosis physician, a biostatistician and an experienced clinical trialist. Episodes of symptomatic recurrent venous thromboembolism, the post-thrombotic syndrome, and death from all causes are reported to the Chair of the Committee on a quarterly basis.

### Ethical considerations

Each patient is provided with written information about the trial and written informed consent is obtained prior to study inclusion. The study protocol has been approved by Health Canada and by the local Research Ethics Committees of the participating centres.

## Discussion

In this report, we describe the protocol of a multicenter randomized placebo controlled trial of elastic compression stockings for the prevention of post-thrombotic syndrome (The SOX Trial). This large, methodologically rigorous study is evaluating the effectiveness and cost-effectiveness of ECS to prevent PTS and has been designed to provide definitive data on the role of ECS in the prevention of PTS. As such, our results will impact directly on the care of patients with DVT. This will also be the first study to prospectively evaluate the predictive role of biomarkers that are reflective of putative underlying pathophysiological mechanisms in the development of clinical PTS (Bio-SOX biomarker substudy). Our findings will increase understanding of PTS and could result in the development of novel therapies aimed at candidate biomarkers.

## Competing interests

The author(s) declare that they have no competing interests.

## Authors' contributions

SRK wrote the initial protocol and designed this study. HS is the trial coordinator and co-authored the manuscript. All other co-authors contributed to study design and/or manuscript revisions.

## Pre-publication history

The pre-publication history for this paper can be accessed here:


